# Modulation of Host Immunity by Human Respiratory Syncytial Virus Virulence Factors: A Synergic Inhibition of Both Innate and Adaptive Immunity

**DOI:** 10.3389/fcimb.2017.00367

**Published:** 2017-08-16

**Authors:** Gisela Canedo-Marroquín, Orlando Acevedo-Acevedo, Emma Rey-Jurado, Juan M. Saavedra, Margarita K. Lay, Susan M. Bueno, Claudia A. Riedel, Alexis M. Kalergis

**Affiliations:** ^1^Millennium Institute on Immunology and Immunotherapy, Departamento de Genética Molecular y Microbiología, Facultad de Ciencias Biológicas, Pontificia Universidad Católica de Chile Santiago, Chile; ^2^Departamento de Biotecnología, Facultad de Ciencias del Mar y Recursos Biológicos, Universidad de Antofagasta Antofagasta, Chile; ^3^Departamento de Ciencias Biológicas, Facultad de Ciencias Biológicas y Medicina, Universidad Andres Bello, Millennium Institute on Immunology and Immunotherapy Santiago, Chile; ^4^Departamento de Endocrinología, Facultad de Medicina, Pontificia Universidad Católica de Chile Santiago, Chile

**Keywords:** hRSV, genes, evasion of host immunity, N protein, immunological synapse

## Abstract

The Human Respiratory Syncytial Virus (hRSV) is a major cause of acute lower respiratory tract infections (ARTIs) and high rates of hospitalizations in children and in the elderly worldwide. Symptoms of hRSV infection include bronchiolitis and pneumonia. The lung pathology observed during hRSV infection is due in part to an exacerbated host immune response, characterized by immune cell infiltration to the lungs. HRSV is an enveloped virus, a member of the Pneumoviridae family, with a non-segmented genome and negative polarity-single RNA that contains 10 genes encoding for 11 proteins. These include the Fusion protein (F), the Glycoprotein (G), and the Small Hydrophobic (SH) protein, which are located on the virus surface. In addition, the Nucleoprotein (N), Phosphoprotein (P) large polymerase protein (L) part of the RNA-dependent RNA polymerase complex, the M2-1 protein as a transcription elongation factor, the M2-2 protein as a regulator of viral transcription and (M) protein all of which locate inside the virion. Apart from the structural proteins, the hRSV genome encodes for the non-structural 1 and 2 proteins (NS1 and NS2). HRSV has developed different strategies to evade the host immunity by means of the function of some of these proteins that work as virulence factors to improve the infection in the lung tissue. Also, hRSV NS-1 and NS-2 proteins have been shown to inhibit the activation of the type I interferon response. Furthermore, the hRSV nucleoprotein has been shown to inhibit the immunological synapsis between the dendritic cells and T cells during infection, resulting in an inefficient T cell activation. Here, we discuss the hRSV virulence factors and the host immunological features raised during infection with this virus.

## Introduction

The human Respiratory Syncytial Virus (hRSV) is one of the most relevant respiratory pathogen affecting infants and the elderly around the world (Lofland et al., 2000; Falsey et al., 2005). Importantly, hRSV infection is considered the major cause worldwide for outpatient visits, hospitalization, and morbidity in children under 5 years of age, during cold seasons (Nair et al., 2010; Lambert et al., 2014; Griffiths et al., 2017). It is estimated that 100% of children at age of 2 have been already infected with hRSV and ~2% of them were hospitalized due to this viral infection (Deshpande and Northern, 2003; Glezen et al., 1986; Hall, 2001). HRSV induces acute lower respiratory tract infections (ALRTIs) with a variety of manifestations, including pneumonia and bronchiolitis, which are accompanied by other symptoms, such as wheezing, cough and respiratory distress (Hall, 2001; Openshaw and Tregoning, 2005). The pulmonary pathology caused by hRSV involves the generation of immunological hyper-responsiveness in the airways (Jafri et al., 2004; Bueno et al., 2008). It has been proposed that an early exposition to hRSV could be associated with reinfections (Dakhama et al., 2005), susceptibility to allergy (Sigurs et al., 2010), recurrent wheezing episodes, also called post-bronchiolitis wheeze (PBW) (Pullan and Hey, 1982; Sigurs et al., 2010), and development of asthma (Pullan and Hey, 1982; Siegle et al., 2010; Wu and Hartert, 2011). Similar to other viruses, hRSV has developed various strategies to avoid the clearance by the host immune system to achieve efficiently spreading through pulmonary epithelial cells (Iannello et al., 2006). The hRSV genome encodes proteins that are fundamental to enter and to infect the host, some of them can be considered as virulence factors (Espinoza et al., 2014). These proteins contribute to the virus strategies to evade the host immune system, in turn improving the viral fitness. In this review article, we discuss the influence of these hRSV proteins in counteracting the host immune response.

## Structural features of hRSV

**Figure 1:**
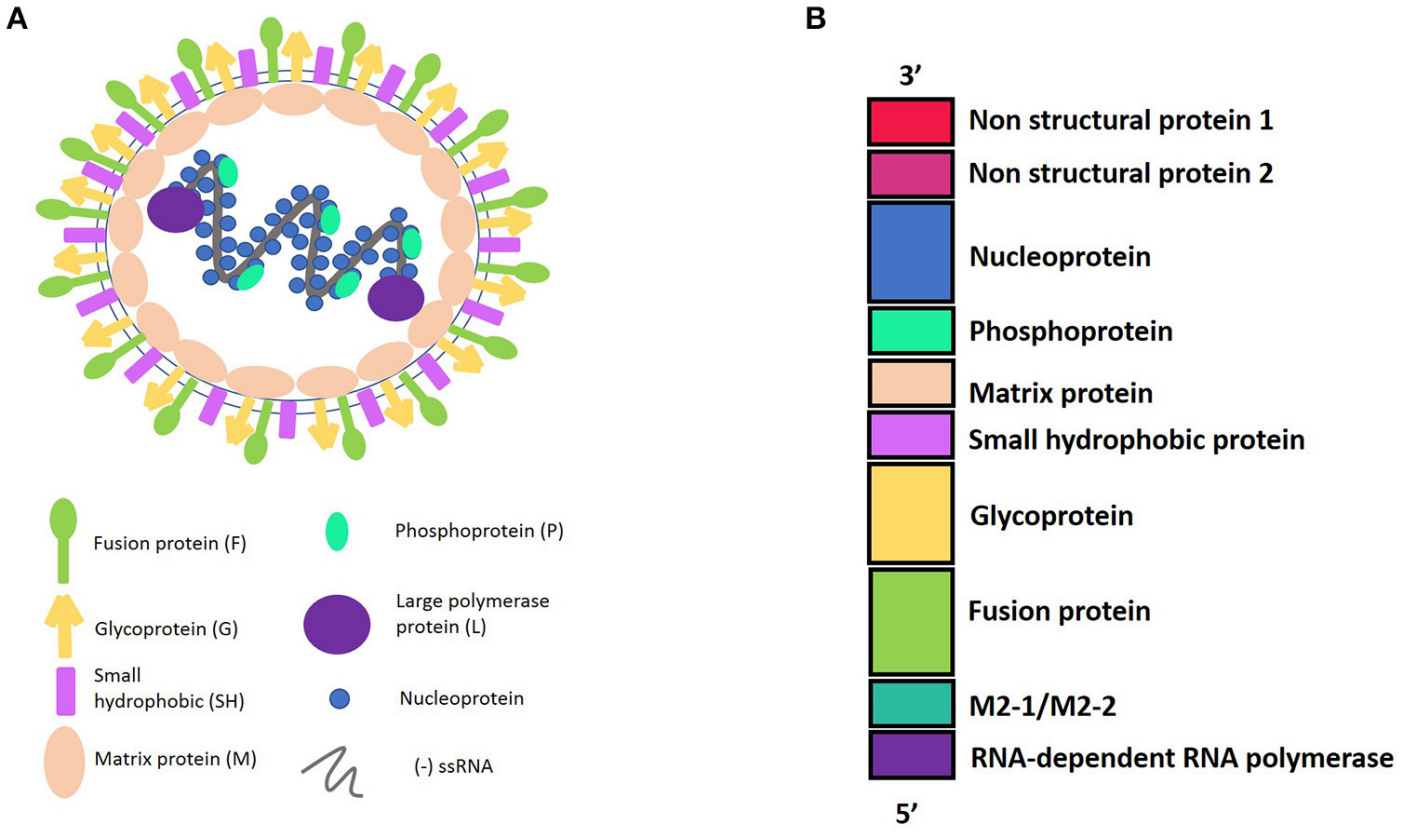
**(A)** Representation of Human Respiratory Syncytial Virus (hRSV) structure and the viral proteins. hRSV proteins have external proteins such as: Fusion protein, Glycoprotein, and Small Hydrophobic protein. The internal proteins are: Matrix protein and proteins belonging to the nucleocapsid, which include the Nucleoprotein, the Phosphoprotein, and the Large polymerase protein. **(B)** HRSV genome harboring the 10 genes, in a 3′–5′ sense, indicating the protein that encode.

HRSV is an enveloped virus, recently characterized as a member of the Pneumoviridae family (Afonso et al., 2016) with a non-segmented genome and negative polarity-single stranded RNA. The 15,200 bp length genome contains 10 genes (3′-NS1-NS2-N-P-M-SH-F-G-M2-L-5′). These genes encode 11 proteins that include from the 3′ to 5′ ends: the non-structural proteins 1 and 2 (NS1 and NS2); the nucleocapsid protein, also known as nucleoprotein (N); another nucleocapsid protein, named the phosphoprotein (P); the matrix protein (M); the small hydrophobic protein (SH); the glycoprotein (G); and the fusion protein (F). In addition, the M2 gene contains two open reading frames, encoding either the M2-1, which is a transcription elongation factor, or the M2-2, which is a regulator of the viral transcription. Finally, the hRSV L gene encodes for the nucleocapsid protein RNA polymerase. Therefore, the hRSV genome encodes nine structural and two non-structural proteins (Collins and Graham, 2008) (Figure 1).

The hRSV non-structural proteins 1 and 2 (NS1 and NS2) are small proteins composed of ~140 amino acids each. The genes encoding for these proteins are located in the three end region of the hRSV genome. Therefore, the transcription of these genes occur in the early stages of the infection (Ling et al., 2008, 2009). Furthermore, it has been described that these proteins are localized in the nuclei and mitochondria of hRSV-infected cells (Swedan et al., 2011). Importantly, NS1 and NS2 proteins impair the induction of the interferon α and β (IFN-α/β) pathways, during the infection of the host (Hastie et al., 2012).

The matrix protein (M) is a non-glycosylated protein that forms a protein layer under the viral envelope, surrounding the nucleocapsid, and interacts with the viral membrane. This protein plays an important role during hRSV replication, budding and assembly (Ghildyal et al., 2002, 2006). Specifically, using a chromatography-multiangle laser light scattering technique, it was found that the M protein is found in a dimeric form in solution, and it is required for budding and viral release (Förster et al., 2015). Furthermore, the M protein interacts directly with the plasma membrane, herein protecting the genome and their components (Money et al., 2009). Both the N and the P, which are nucleocapsid proteins, are essentials for the replication and transcription activities. Indeed, the N protein is a 43 KDa protein (Cannon, 1987) that contains an helical structure bound to the ribonucleoprotein complex. Furthermore, the N protein covers the viral RNA, during transcription and replication of the virus in the host cell, thereby contributing in protecting it from the antiviral response, and in preserving the structure of the ribonucleoprotein complex (Bhella et al., 2002; Tawar et al., 2009). In addition, the N protein has been shown to impair the immunological synapse between dendritic cells and T cells (Cespedes et al., 2014). Interestingly, the N protein was found to be present on the surface of hRSV-infected cells (Cespedes et al., 2014). However, further studies are required to elucidate the exact mechanism in how this event occur. On the other hand, the P gene encodes for a protein of 241 amino acids, which is a co-factor of the hRSV L-polymerase. Moreover, it binds the N protein with the helical structure, forming a specific P-N complex (Bakker et al., 2013). Besides, the P protein can work as a chaperone, preventing the interaction of the newly synthesized unassembled N protein with the viral RNA, during the encapsidation process of the new hRSV virions in infected cells. In addition, the P protein participates in hRSV replication and transcription events (Galloux et al., 2012, 2015). On the other hand, the L gene contains a highly conserved sequence that encodes for a 2,165 amino acid long protein that works as the major catalytic subunit of the RNA-dependent RNA polymerase (RdRp) complex (Fix et al., 2011). Also, the M2-1 and the M2-2 proteins participate and regulate the hRSV transcription and replication processes. While the M2-1 along with the P protein regulate the transcription of mRNAs, contributing to the processivity of the L-polymerase; the M2-2 protein mediates the transition from transcription to replication (Bermingham and Collins, 1999; Collins et al., 1995; Cheng et al., 2005). Recently, it was shown that the M2-1 is located between the ribonucleoprotein complex and the M protein in the viral particle (Kiss et al., 2014).

The F protein is a transmembrane protein composed of 574 amino acids (McLellan et al., 2013) that is required for hRSV fusion and entry to the host cell. Further, the F protein promotes viral fusion to the host cell membrane and formation of epithelial cell-cell syncytium in the infected airway tissue (Openshaw and Tregoning, 2005). Such function has been previously suggested through the binding of two specific inhibitors of hRSV F protein (CL-309623 and RFI-641), herein preventing the entry and fusion of the virus to the Vero cells (Razinkov et al., 2002). Another study, in which a mutant hRSV strain, lacking the glycoprotein (G) and SH genes, was studied and showed that the F protein favors the post-attachment rate entry in the Hep-2 cells. Thus, the presence of F protein is required for hRSV entry into the host cell (Techaarpornkul et al., 2001). Furthermore, it has also been reported that the F protein interacts with nucleolin, an ubiquitous host receptor (Losfeld et al., 2009; Tayyari et al., 2011). Specifically, co-immunoprecipitation assays, using human airway epithelial cell lysates, showed that the nucleolin interacted with the F protein (Tayyari et al., 2011). The requirement of this receptor for the entry of hRSV to cells was demonstrated by applying a treatment with an anti-nucleolin antibody, which caused a poor co-localization of the virus on the cell surface and decreased hRSV infection (Tayyari et al., 2011). Recently, it was found that the hRSV entry is mediated by the Rab5 macropinosomes, since Rab5-deficient HeLa cells displayed lower levels of hRSV infection, as compared to wild type cells (Tayyari et al., 2011). Further, such hRSV entry mechanism was demonstrated to be pH, clathrin and dynamin independent (Tayyari et al., 2011). Moreover, it was shown that hRSV transient macropinocytosis requires two cleavages of the F protein (Krzyzaniak et al., 2013). Nevertheless, this notion is at variance with a previous study, proposing that the hRSV entry takes place due to a clathrin-mediated endocytosis (Kolokoltsov et al., 2007).

The hRSV G is a glycosylated transmembrane protein of 80 kDa (Melero et al., 1997; McLellan et al., 2013). Moreover, a soluble form of the G protein, lacking the cytoplasmatic and transmembrane domains, has been described to work as a decoy for neutralizing antibodies, thereby avoiding the antiviral response of the host (Bukreyev et al., 2008, 2012). Additionally, the G protein, due to its sequence diversity, has been employed as a molecular marker to discriminate between the hRSV subtypes A and B (Anderson et al., 1985; Mufson et al., 1985). Importantly, the G gene encodes for a viral transmembrane protein that is important for the hRSV attachment to the host cell (Levine et al., 1987). In fact, the attachment function of the G protein was first identified in HeLa cell monolayers (Levine et al., 1987). These cells were treated with anti-G and then challenged with a radiolabeled hRSV. As a result, an inhibition of viral particle attachment to the HeLa cells was observed (Levine et al., 1987). Initially, it was thought that the G protein binds to the heparan sulfate on the extracellular matrix of the host cells (Feldman et al., 1999). However, a study in human airway epithelium (HAE) showed that CX3R1, a receptor on ciliated cells, is pivotal for the G protein-mediated attachment of viral particles (Johnson et al., 2015). Indeed, a decreased of hRSV infection was observed with the neutralization of CX3R1 by using two specific monoclonal antibodies (Johnson et al., 2015). The molecular explanation for this specific attachment of the virus with CX3R1, derives from the similarity of the G protein to CX3CL1, a chemokine produced by immune cells, such as macrophages and dendritic cells (Papadopoulos et al., 1999). The sequence similarity between the G glycoprotein and the CX3CL1 molecule locates at the chemokine-like motif, composed of 4 amino acids, at the positions 182–186 of the G protein conserved region (Tripp et al., 2001; Harcourt et al., 2006).

Moreover, the SH gene encodes a 64 amino acid protein for hRSV serotype A and a 65 amino acid protein for the hRSV serotype B (Collins and Mottet, 1993). The SH glycoprotein is neither required for the entry to the host cells nor needed for viral RNA replication (Bukreyev et al., 1997; Techaarpornkul et al., 2001). However, several studies that evaluated mutant hRSV strains that lack the SH gene, have shown an attenuation in the virulence (Karron et al., 1997; Techaarpornkul et al., 2001; Rixon et al., 2004). Also, in other studies the hRSV SH protein is suggested to prevent apoptosis in hRSV-infected cells (Lin et al., 2003; Fuentes et al., 2007). Specifically, these studies demonstrate that SH protein has an important role in hRSV infection, since SH prolongs the cellular life, allowing a continuous virion production. Recently, the SH pentameric model shows that the structure anchored in a lipid bilayer, display N-terminal and C-terminal ends, which are composed by random coil and β-sheet structures, conforming a pore structure (Araujo et al., 2016). Those regions are suggested to be important channels for hRSV pathogenesis. Thereby, it may be a mechanism to inhibit ligand-mediated apoptosis in the hRSV-infected cells.

## Effect of hRSV proteins in the innate immune response: hRSV recognition by the host and interference of the IFN pathway

Different cellular receptors recognize hRSV molecular patterns, including Toll-like receptors (TLR), trigger an innate immune response. Interestingly, an interaction between the F protein and the TLR-4 activate this receptor, herein initiating an antiviral immune response (Rallabhandi et al., 2012). Along these lines, an hRSV persistent infection was found in TLR-4 deficient mice, as compared to wild-type mice (Kurt-Jones et al., 2000). Thus, TLR-4 activation is clearly important for an appropriate antiviral response, avoiding a persistent hRSV infection (Kurt-Jones et al., 2000).

The type I IFN response is key for the antiviral response (García-Sastre and Biron, 2006). In this regard, NS1 and NS2 proteins hamper the IFN-α/β production during hRSV infection (Spann et al., 2004, 2005). The function of these proteins was discovered in human airway epithelial cells (A549) and human peripheral blood monocyte cell derived-macrophages, which were infected with the hRSV A2 strain and the recombinant hRSV mutants, lacking the NS1 gene (ΔNS1), the NS2 gene (ΔNS2) and both NS1 and NS2 (ΔNS1/NS2). Specifically, in this study, the authors observed an increase in IFN-α/β production in cultures infected with the ΔNS1/NS2 hRSV strains (Spann et al., 2004). Furthermore, it has been reported that the NS1 protein co-localizes with a critical molecule of the type I IFN pathway, the mitochondrial signaling protein (MAVS), thus inhibiting the phosphorylation of the interferon regulatory factor 3 (IRF-3) (Boyapalle et al., 2012). Likewise, NS1 can bind directly to IRF-3 and the CREB binding protein (CBP), both transcriptional activators of the type I IFN system. Therefore, it is thought that those interactions reduce the binding between IRF-3 and the IFN-β promoter, decreasing the antiviral cascade activation (Ren et al., 2011). Similarly, NS2 interferes with the activation of the IRF-3 by binding to RIG-I (Ling et al., 2009). In addition, NS1 has been shown to promote the proteosomal degradation of 2′–5′Oligoadenylate synthetase-like protein (OASL), which is a type I IFN-inducible effector molecule that control viral replication (Dhar et al., 2015). Moreover, it was suggested that the NS1 protein increases miR-29a levels, which inhibits the expression of the IFNα/β receptor (IFNAR1) that is essential for the type I IFN function (Zhang et al., 2016). Following the IFNAR1 mRNA interference by hRSV, the NS1 protein suppresses the expression of miR-24, by producing the Kruppel-like factor 6 (KLF6). Specifically, KLF6 induces the production of TGF-β, which in turn inhibits miR-24 and thought to cause cell arrest, reducing apoptosis and increasing viral replication (Bakre et al., 2015). This notion is supported by previous studies showing that NS1 and NS2 were involved in the inhibition of cell apoptosis in the early stages of the hRSV infection (Bitko et al., 2007). Thus, through the action of NS proteins, hRSV can inhibit apoptosis to persist for longer periods in the host and avoid the innate immune response.

Additionally, it has recently been shown that both the NS1 and NS2 proteins induce the proteosomal degradation of the signal transducer and activator of transcription 2 (STAT2) (Whelan et al., 2016). As a result, the host antiviral response mediated by the type IFN I pathway is largely impaired, as well as viral clearance (Lo et al., 2005; Ramaswamy et al., 2006; Elliott et al., 2007; Jie et al., 2011). Moreover, it was reported that BALB/c mice infected with recombinant hRSV lacking the NS genes (hRSVΔNS1, hRSVΔNS2, and hRSVΔNS1/NS2) showed an increased frequency of cytotoxic T cells (CTLs) as compared to mice infected with wild-type hRSV, which are susceptible to suppression of the type I IFN response (Kotelkin et al., 2006). Therefore, hRSV NS proteins are critical for hRSV pathogenesis not only by inhibiting the type I IFN pathway but also by impairing the proliferation of host CTLs. The capacity of hRSV to inhibit this process is likely to contribute to the frequent re-infections caused by hRSV throughout life.

Another important protein that affects the ability of the innate immune response to recognize and respond to an hRSV infection is the G protein, specifically a CX3C motif present in this protein (Chirkova et al., 2013, 2015). A recent study in human airway epithelial cells infected with hRSV (with an intact CX3C motif) or with a strain with a mutant motif (CX4C) produced lower levels of IFN-γ and IFN-λ, as compared to IFN-α, suggesting that CX3CR is playing a suppressor role in the IFN pathways (Chirkova et al., 2013). The molecular similarity between the G protein and the CX3CR1, as above mentioned, impairs the binding of the CX3CL1 chemokine to the CX3CR1 receptor, promoting hRSV infection in the host (Chirkova et al., 2013, 2015). Further, it has been evidenced the presence of CX3CR1 not only in airway epithelial cells, but also in NK and cytotoxic T cells (Mionnet et al., 2010; Johnson et al., 2015). Thus, the G protein of hRSV also contributes to impairing the host immune response and promotes viral dissemination.

Apart from triggering IFN responses, hRSV activates the inflammasome through the activity of the viral SH protein. It was observed that the inflammasome activation and IL-1β secretion in hRSV-infected primary human lung epithelial cells was higher than in cells infected with a recombinant hRSV, lacking the SH protein (hRSVΔSH) (Triantafilou et al., 2013; Russell et al., 2015). Also, the hRSV-infected cells express caspase 1, a pivotal element in the inflammasome activation (Triantafilou et al., 2013). In addition, a decrease in IL-1β production was found in lung epithelial cells, treated with ion channels inhibitors (hexamethylene amiloride, EIPA and benzamil) upon hRSV infection (Triantafilou et al., 2013). Taken together, these data suggest that hRSV SH protein is involved in the assembly of ion channels required for the inflammasome activation (Gan et al., 2008, 2012; Triantafilou et al., 2013). However, additional research is needed to elucidate the mechanisms employed by the SH protein to impair the proper function of the host immune system.

## Effect of hRSV proteins in the adaptive immune response: inhibition of the immunological synapse and the T cell activation

Antigen-presenting cells (APCs) play an essential role during the infection, as these cells can present peptide-, lipid-, and vitamin B precursor-based antigens (Ags) in the context of the major histocompatibility complex (MHC) or MHC-like molecules, respectively (Beckman et al., 1994; Germain, 1994; Kjer-Nielsen et al., 2012). The pMHC complexes on the surface of APCs are recognized by the T cell receptor (TCR), which is an antigen-specific molecule expressed on the surface of T cells. Thus, the naïve T cell becomes activated contributing to the initiation of the adaptive immune response against pathogens (Brownlie and Zamoyska, 2013). Such interaction occurs through the variable regions of the TCR α and β chains. At the interface, between pMHC complexes and the T cell receptor, there are three hyper variable loops, termed complementarity-determining regions (CDRs) that make contact with antigens (Hughes et al., 2003). Effective interaction between TCR and pMHC molecules triggers a tyrosine phosphorylation cascade inside of the T-cell membrane, as well the activation of multiple signaling pathways (Janeway et al., 2001). The adaptive immune response complex processes, such as the T cell proliferation, require of the TCR engagement and triggering of the respective signaling cascades, which occur within minutes or hours. However, the sustained TCR engagement with its cognate antigen that takes place is constrained by many barriers. As a first point, TCR engagement is limited by the small size of the TCR and MHC molecules, surrounded with large and abundant glycoproteins like CD43 and CD45, that imposes steric hindrance to TCR-MHC interaction (Cyster et al., 1991; McCall et al., 1992). On the other hand, the affinity of TCR for peptide-loaded MHC molecules is low (<100 μM) (Davis et al., 1998; Knapp et al., 2015) and the continuous movement of T cells makes sustained recognition of pMHCs by TCRs a challenging process.

Different studies related to the interaction between pMHCs with TCRs have revealed the formation of a specialized supra molecular structure, termed the immunological synapse (IS). The formation of this interaction requires the engagement of TCR by pMHCs. This process triggers the rearrangement of a variety of receptor-ligand pairs. Firstly, the interaction between LFA-1 and ICAM-1 proteins anchors the central region of the nascent IS (Grakoui et al., 2015). This interaction facilitates cytoskeletal movements that ultimately can position peripheral TCRs close to their cognates ligands. This rearrangement produces TCR-enriched migrating regions known as TCR microclusters. In the subsequent steps, the TCR microclusters are arranged in the central region of the IS in a domain known as the central supramolecular activation cluster (cSMAC) (Grakoui et al., 1999; Varma et al., 2006). Concomitantly, ICAM-1-LFA1 pairs are positioned into a peripheral adhesion domain or pSMAC, leading to assembly of the IS (Grakoui et al., 1999).

As the assembly of the IS is a pivotal requirement for the establishment of proper T-cell responses and the development of the T-cell memory, many pathogens such as viruses have developed virulence mechanisms to impair the assembly of the IS and the initiation of the adaptive immunity (Muller et al., 2006; Thoulouze et al., 2006). Specifically, our research group has studied that hRSV infection promotes DCs maturation and the secretion of IL-6 and IL-10. However, these APCs are impaired in their ability to induce T cell activation despite the fact that these cells present either allo- cognate or superantigens to T lymphocytes (Gonzalez et al., 2008). Remarkably, hRSV suppression of T cell activation by using DCs *in vitro* was not mediated by inhibitory soluble factors secreted by these APCs. By contrast, it was found that stimulating cytokines such as IL-2 were increased in the supernatants obtained from hRSV-infected DCs.

Interestingly, human monocyte-derived myeloid dendritic cells infected with recombinant hRSV, lacking the NS genes (hRSVΔNS1 and hRSVΔNS2), showed a significantly increased expression of maturation markers, as compared to wild-type hRSV-infected cells (Munir et al., 2008). Furthermore, virus lacking the NS1 protein (ΔNS1) induced an increased proliferation and activation of CD8^+^ T cell specific to epithelial cells (Munir et al., 2011). These results suggest that the NS genes modulate the maturation of DCs, reduce antigen presentation and the T cell activation, impairing key components of the host immune response against hRSV infection.

Remarkably, it has been suggested that hRSV proteins impair the assembly of the IS between T cells and DCs (Gonzalez et al., 2008). As supporting evidence for this hypothesis, it was found that polarization of the Golgi apparatus was reduced to a background frequency in OT-II T cells pulsed with OVA peptide and co-cultured with hRSV-infected DCs. Thus, the impairment of the T cell function by hRSV infection seems to be a process that requires cell-to-cell contact. Moreover, it was shown that impairment of the T cell function by hRSV could be a consequence of an anergic-like phenotype of T-lymphocytes triggered by a dysfunctional IS assembly between T cells and DCs, since T cells co-cultured with hRSV-infected DCs remained irresponsive to a strong activating stimulus, such as of an anti-CD3ε antibody treatment (Gonzalez et al., 2008). We have also recently found that the hRSV-N could be involved in inhibiting T-cell activation during infection (Cespedes et al., 2014). Indeed, experiments on supported lipid bilayers (SLBs), loaded with pMHC complexes, showed that the hRSV N protein prevented the IS assembly in both naive CD4^+^ T cells and, to a lesser extent, antigen-experienced T-cells. These results are attributable to an increased sensitivity of the antigen-experienced T-cell to pMHCs, as the frequencies of the mature IS in antigen-experienced cells were significantly increased compared to those observed in naïve T-cells (Cespedes et al., 2014). This result, could be explained because antigen experienced T-cells can form large sizes of TCR oligomers, which showed increased pMHC sensitivity compared to shorter TCR oligomers found in naïve CD4^+^ T cells (Kumar et al., 2011).

As a general point, inhibition of the IS assembly by the action of the N protein, observed in this work, was in part attributable to diminished TCR signaling and pMHC clustering at the T-cell- bilayer interface. This criterion was not matched by proteins that do not inhibit the IS, including hRSV M2-1 and GFP. Interestingly, it was found that soluble N protein does not colocalize with pMHCs loaded on SLBs. Instead, the N protein interacts in trans with the TCR molecules of T-lymphocytes, as TCR signal colocalizes with fluorescent-tagged N proteins.

**Figure 2:**
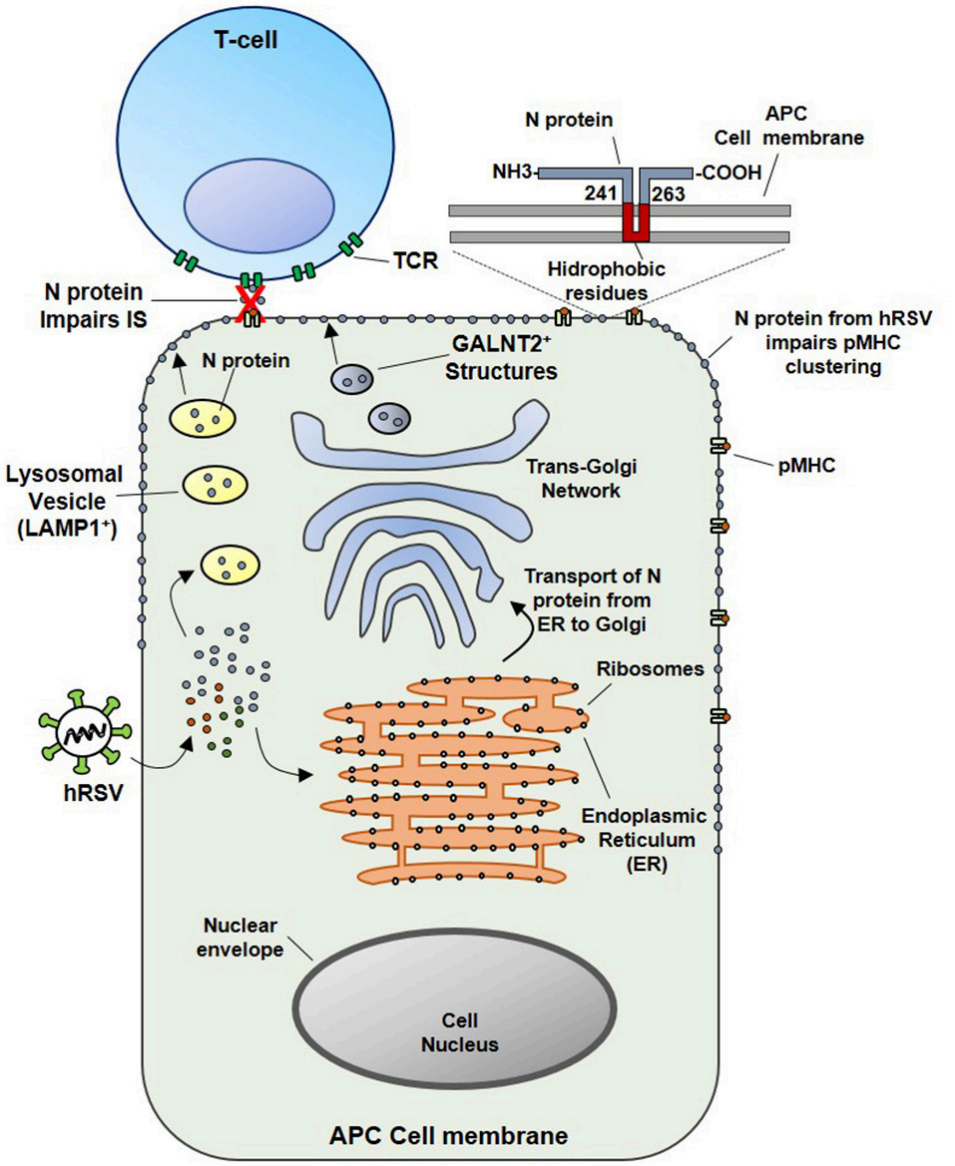
Proposed model for the inhibition of the IS assembly between Antigen Presenting Cells (APCs) and T lymphocytes, triggered by N protein. At an earlier infection stage point (such as 24 hpi), attachment and fusion of hRSV to APC membranes is enough to deliver N protein in the cytosol of an infected APC. Subsequently the N protein could be transported to the cell surface through the lysosomal exocytic pathway (LAMP1^+^ structures, in yellow) or the trans-Golgi network (GALNT2^+^ structures shown in blue). As the N protein is expressed on the surface of APC, the clustering of pMHC molecules is inhibited, as well as, the IS assembly. Based on primary amino acidic sequence analysis, we propose that a hydrophobic-rich residue region of the hRSV N could serve to anchor the N protein on the APC surface membrane (inset). Numbers indicate the exact positions in where this region is localized in the N protein, according to TMHMM Server v. 2.0 analysis.

Since the soluble N protein colocalizes with the TCR, it was hypothesized that hydrophobic residues within the N protein could transiently interact with the phospholipid bilayer after reaching the membrane of infected cells. In this sense, this study also demonstrated that N-protein, which is associated with viral RNA, could be expressed on the surface of viral particles as an early event (1 h) post-infection (pi). Importantly, the results obtained also suggest that virus attachment and virus-cell fusion to target cells would be sufficient to deliver the N protein on the APCs surface to impair the IS (Cespedes et al., 2014). However, the specific mechanisms accounting for the transport of the N protein to the cell surface are not well-understood. Nevertheless, we suggest that the N protein could interact with the Golgi apparatus or lysosomal membranes in the cytosol of infected cells (Figure 2). These notions are supported by the observations that at 24 h pi, a time point in which infected cells expressed mostly soluble N-protein (Fearns et al., 1997), the hRSV-N colocalizes with a membrane-anchored Golgi enzyme GALNT2^+^ structures. Further, it was shown that treatment with Brefeldin-A, an inhibitor of protein transport from the endoplasmic reticulum to the Golgi apparatus (Klausner et al., 1992), reduces the amount of N protein on the surface of infected cells and triggers its accumulation in GALNT2^+^ and Lysosomal-associated membrane protein 1 (LAMP1^+^) compartments. Therefore, both trans-Golgi and lysosomal exocytic pathway could be involved in the transport of the N protein to the cell surface (Cespedes et al., 2014) (Figure 2).

## Concluding remarks

HRSV is one of the major respiratory pathogens causing a major health burden worldwide. This virus can produce several reinfections throughout life even can leave sequelae, such as asthma and respiratory allergies. Research on hRSV has taken more than five decades, however, up to date, there are no licensed vaccines available to combat this virus. Currently, only an expensive prophylactic treatment is available based on the administration of specific monoclonal antibodies against the F protein (e.g., Palivizumab). HRSV has co-evolved with humans during years and as a result, different hRSV genes are considered as virulence factors, which contribute to hRSV survival and dissemination in the host. Furthermore, some of these genes have the capacity to interfere with the assembly of the IS, interfering with the establishment of a proper T cell memory, favoring herein hRSV reinfections.

## Author contributions

GC, OA, ER, and AK wrote the manuscript; AK, JS, CR, ML, and SB reviewed the manuscript. All authors listed approved the version to be published and have made a substantial and intellectual contribution to the work.

### Conflict of interest statement

The authors declare that the research was conducted in the absence of any commercial or financial relationships that could be construed as a potential conflict of interest.
